# Effects of Test Length and Scoring Methods on Pass or Fail Classification Accuracy in Single-Choice Examinations: Simulation Study

**DOI:** 10.2196/98358

**Published:** 2026-10-15

**Authors:** Christiane Pink, Yola Meisel, Arvid Vetter, Philipp Kanzow

**Affiliations:** 1 Department of Restorative Dentistry, Periodontology and Endodontology, University Medicine Greifswald, Fleischmannstr. 42, Greifswald, 17475, Germany, 49 3834 867130, 49 3834 867171

**Keywords:** assessment, classification accuracy, educational assessment, educational measurement, examination length, measurement error, Monte Carlo simulations, multiple-choice examination, pass or fail decisions, scoring, scoring system, single-response, test length, testing

## Abstract

**Background:**

Multiple-choice examinations are widely used in dental and medical education. To reduce the effects of random guessing, several scoring methods incorporating penalty scores have been proposed. However, it remains unclear whether such scoring methods improve the accuracy of pass or fail decisions when pass marks are appropriately calibrated.

**Objective:**

This study aimed to examine pass or fail classification accuracy under 3 scoring methods (dichotomous scoring, formula scoring, and right-minus-wrong scoring) in summative examinations using single-choice Type A items with 5 answer options and to evaluate the effects of test length and the distribution of the model parameter *k* (knowledge-based response probability).

**Methods:**

Monte Carlo simulations and analytical derivations were used to evaluate examinations with varying test lengths of up to 300 items. Pass marks were calibrated to the same model-defined cutoff for all scoring methods. Classification accuracy, sensitivity, specificity, and misclassification rates were analyzed across different distributions of *k*.

**Results:**

With every item answered and pass marks calibrated to the same model-defined cutoff, the 3 scoring methods produced identical pass or fail classifications, as expected from their linear relationship. Classification accuracy increased with test length but was strongly influenced by the distribution of *k*. Under a uniform distribution, mean accuracy reached 0.90 after 24 items and 0.95 after 94 items, whereas substantially longer tests were required when *k* was concentrated near the cutoff. These item numbers are model- and distribution-dependent and should not be interpreted as recommended examination lengths. Misclassification was highest near the decision threshold.

**Conclusions:**

Under the modeled conditions of complete responding, calibrated pass marks, and unchanged response behavior, the investigated scoring methods yielded identical pass or fail classifications. Classification accuracy increased with test length and depended strongly on the distribution of *k*, with greatest uncertainty near the pass or fail cutoff. These findings apply to the structural effects of the scoring transformations examined here and should not be generalized to behavioral effects of scoring rules in real examinations.

## Introduction

Knowledge tests using multiple-choice items have been used for over a century [1,2] and are widely applied in medical and dental education. Both university-level examinations and nationwide licensing examinations (eg, the United States Medical Licensing Examination) use multiple-choice formats. Single-choice items with 3 to 6 answer options are the most common item format [3].

A longstanding criticism of multiple-choice examinations is the possibility of obtaining correct answers through guessing. Observed responses reflect a combination of knowledge-based response probability (*k*) and stochastic guessing processes. To address this issue, numerous scoring methods have been proposed to approximate examinees’ *k* by correcting for guessing. These methods apply penalty scores (ie, malus points) to incorrect responses. A recent systematic review identified 21 different scoring methods for single-choice items, resulting in scores ranging from –3 to +1 points per item [4].

Despite their widespread use, penalty-based scoring methods are often assumed to improve the accuracy of pass or fail decisions, although empirical evidence supporting this assumption remains limited. However, such scoring methods may introduce construct-irrelevant variance and thereby negatively affect test validity [5]. Specifically, they may introduce construct-irrelevant variance related to examinees’ personality, such as risk-taking behavior, and test-wiseness (ie, strategic response behavior unrelated to knowledge) [6]. Additionally, such scoring methods may be perceived as unfair or discouraging by examinees [7], and in some countries, legal regulations restrict the use of scoring methods that allow negative scores for individual items [8].

Summative examinations are designed to differentiate between examinees who pass and those who fail, commonly based on a minimum *k* of 50% [9]. Although a 50% threshold is often used as a conceptual reference point, the present model is independent of the specific cutoff and can be generalized to other thresholds. High reliability of examination results is essential to minimize false-positive outcomes (ie, examination scores at or above the pass mark despite *k*<0.5) and false-negative outcomes (ie, examination scores below the pass mark despite *k*≥0.5).

Although scoring methods involving penalty points remain controversial, it remains unclear whether they improve the accuracy of pass or fail decisions when pass marks are properly calibrated. Although invariance under linear score transformations is well established in psychometric theory, its implications for pass or fail classification accuracy have not been explicitly quantified under calibrated decision thresholds in applied assessment contexts. Therefore, this study examines the effects of scoring methods and test length in single-choice examinations. It isolates the structural (ie, mathematical) effects of scoring transformations from the behavioral effects of examinees to evaluate whether scoring methods can improve pass or fail classification accuracy. Specifically, it assesses the purely mathematical contribution of scoring transformations to classification accuracy under explicitly defined modeling assumptions.

## Methods

### Ethical Considerations

This study was based exclusively on computer-generated simulated data and did not involve human participants, identifiable human material, or identifiable human data. Therefore, research ethics committee review and informed consent were not applicable.

### Study Design and Scoring Methods

This simulation study investigated single-choice Type A items with 1 correct answer option and 4 distractors. The focus on 5 answer options reflects common practice in medical education; however, the analytical model can be generalized to other numbers of options with adjusted guessing probabilities. The following 3 scoring methods most frequently described in the literature were selected [4]:

1. Dichotomous scoring (also known as number-right scoring or raw score), awarding 1 credit point for a correct response and 0 points otherwise
2. Formula scoring (also known as the correction for guessing formula or corrected score), awarding 1 credit point for a correct response and deducting 0.25 credit points (1/[*n*–1]) for each incorrect response [10]
3. Right-minus-wrong scoring (also known as the R–W method), awarding 1 credit point for a correct response and deducting 1 credit point for each incorrect response [2]

For each scoring method, the *expected chance scores* from random guessing [11] and cutoff scores corresponding to 50% knowledge-based response probability were derived analytically and are summarized in Table 1.

**Table 1.** *Expected chance scores* from random guessing and cutoff scores corresponding to 50% knowledge-based response probability (*k*=0.5) for single-choice Type A items with 5 answer options.

Scoring methods	Expected chance score [4,11]	Cutoff score at *k*=0.5 (%)^a^
Dichotomous scoring	0.20	60
Formula scoring	0.00	50
Right-minus-wrong scoring	–0.60	20

a Expressed as a percentage of the maximum possible score.

### Simulation Model

Examination data were generated using a Monte Carlo simulation framework designed to isolate the mathematical effects of scoring transformations. Monte Carlo simulation is a well-established approach for evaluating statistical properties of measurement procedures [12]. Each examinee was characterized by a knowledge-based response probability *k*∈[0,1], representing the assumed probability of producing a correct response based on knowledge rather than guessing. This definition is consistent with the probabilistic framework used in previous work on scoring methods [13]. The parameter *k* is a model parameter and should not be interpreted as an observed percentage of knowledge, a direct measure of competence, or a validated latent ability measure.

For a given value of *k*, the probability of answering an item correctly was modeled as p(*k*)=*k*+(1–*k*)/5, representing a combination of knowledge-based responding (with probability k) and uniform random guessing (with probability 1−*k* across 5 response options). The modeling approach serves as a simplified baseline that does not include informed guessing or partial knowledge. It is conceptually related to probabilistic frameworks used in item response theory, in which observed responses are linked to underlying examinee characteristics [14]. The formulation assumes that lack of knowledge results in random guessing across all answer options, excluding informed guessing or distractor elimination strategies, consistent with established models of multiple-choice responding that include a guessing component [15]. Responses to individual items were assumed to be conditionally independent given *k* and were generated as Bernoulli trials with success probability p(*k*) [16]. The model is intended to isolate the structural effects of scoring transformations rather than to represent the full complexity of response behavior in real examinations.

### Simulated Examinee Groups

To represent different levels of knowledge-based response probability, 3 groups of examinees were selected for illustrative graphical representation: examinees with (1) low *k* (*k*=0.25; expected to fail), (2) intermediate *k* (*k*=0.55; expected to pass), and (3) high *k* (*k*=0.85; expected to pass). These values were chosen as illustrative model scenarios.

### Scoring and Pass or Fail Classification

For each simulated examination, response data were scored according to the 3 scoring methods. In a second approach, total scores were converted into proportions of the maximum possible score to ensure comparability among varying test lengths. For each scoring method, a pass mark (ie, the expected score of an examinee with *k*=0.5) was defined (Table 1). This calibration is to ensure comparability across scoring methods and to isolate the effect of score transformation. Examinees with observed scores at or above the pass mark were classified as passing, whereas those below the pass mark were classified as failing. Because the number of correct responses is discrete, noninteger pass thresholds were rounded up to the next attainable score. A small numerical tolerance was applied to avoid floating-point artifacts.

### Outcome Measures

The primary outcomes were classification accuracy and misclassification rates, analyzed in relation to item number, scoring method, and distance from the model-defined cutoff. Classification accuracy was defined as the proportion of examinees correctly classified as passing or failing relative to the model-defined cutoff at *k*=0.5.

For each simulation, 4 classification outcomes were identified:

- True positives (TPs): *k*≥0.5 and classified as passing
- False positives (FPs): *k*<0.5 and classified as passing
- True negatives (TNs): *k*<0.5 and classified as failing
- False negatives (FNs): *k*≥0.5 and classified as failing

Overall accuracy was then computed as (TP+TN)/(TP+FP+TN+FN).

### Statistical Analysis

All analyses were conducted using R (version 4.5.2; R Foundation for Statistical Computing) [17]. Simulations were implemented using custom scripts. Detailed model specifications, analytical equations, selected implementation code, and the fixed random-number settings used for stochastic analysis component are provided in Multimedia Appendix 1. Machine-readable study-specific outputs underlying the reported tables and figures and the software session information are provided separately in Multimedia Appendices 2 and 3.

To illustrate variation in examination scores with increasing test length, cumulative score trajectories were generated for randomly selected examinees from each of the 3 predefined knowledge-based response probabilities (*k*=0.25, 0.55, and 0.85) and plotted as functions of the number of administered items.

To evaluate classification performance, simulations with 10,000 replications were conducted using cohorts of 20 to 1000 examinees to ensure numerical stability of the results. Importantly, cohort size does not influence the classification mechanism itself but only the precision of estimated accuracy metrics. Corresponding simulation-based variability bands were graphically displayed for 50, 100, and 1000 examinees. Two different distributions of knowledge-based response probability were considered: (1) a uniform distribution over the interval [0,1], representing a heterogeneous population with uniformly distributed *k*; and (2) a truncated normal distribution centered at the cutoff *k*=0.5, with approximately 95% of values falling between *k*=0.05 and *k*=0.95, representing a population concentrated around the cutoff threshold.

For each cohort, examination responses were generated and scored according to the 3 scoring methods. Classification accuracy, sensitivity, and specificity were computed as functions of test length. In addition to the simulation-based estimates, theoretical classification accuracy was derived analytically from the underlying stochastic response model using binomial probabilities and normal approximations. The analytical results were compared with the corresponding simulation estimates to assess numerical consistency and convergence [18].

## Results

Simulation-based estimates were numerically consistent with the corresponding analytical results across all examined conditions.

### Illustrative Score Variation Across Increasing Item Numbers

Figures 1 and 2 illustrate variation in examination scores with increasing item numbers. At low item counts, substantial fluctuations in trajectories of cumulative scores and the percentage of the maximum possible score were observed. With increasing examination length, interindividual variability decreased and trajectories converged toward their expected values. Beyond a certain number of items, relative examination results changed only marginally with the addition of further items. Across all scoring methods, convergence toward the expected score occurred earlier for higher *k* (*k*=0.85) than for intermediate (*k*=0.55) and low *k* (*k*=0.25). This pattern is consistent with a reduced contribution of guessing-related random variation at higher *k*.

**Figure 1.**
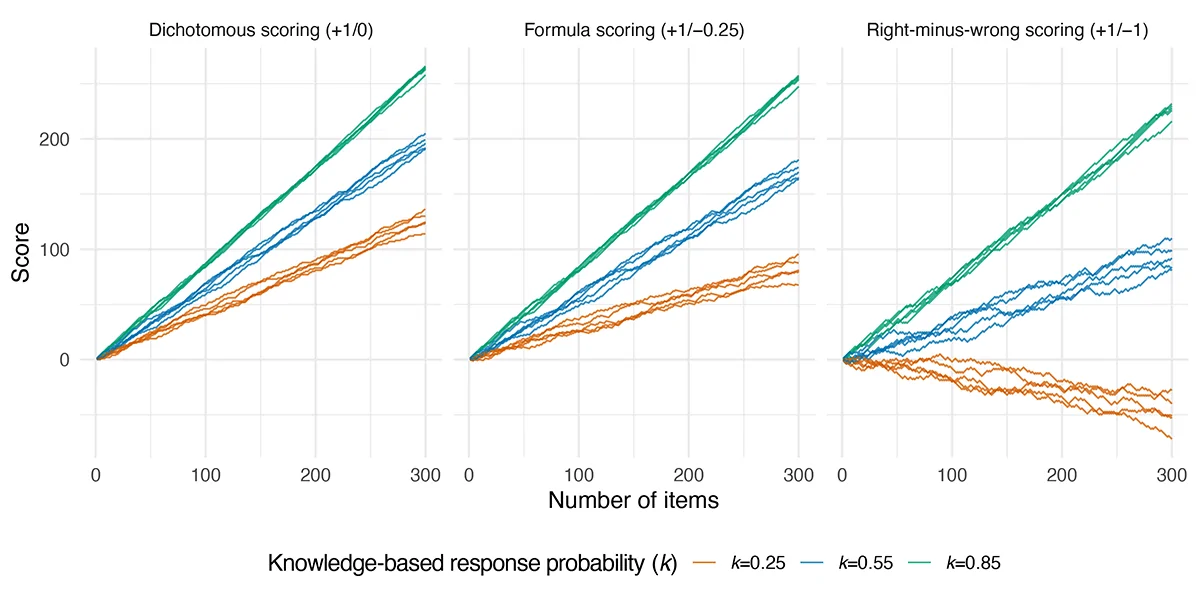
Convergence of total score trajectories with increasing item numbers. From a simulated cohort, 5 exemplary examinees were selected for different knowledge-based response probabilities (*k*=0.25, 0.55, and 0.85) and plotted for each scoring method.

**Figure 2.**
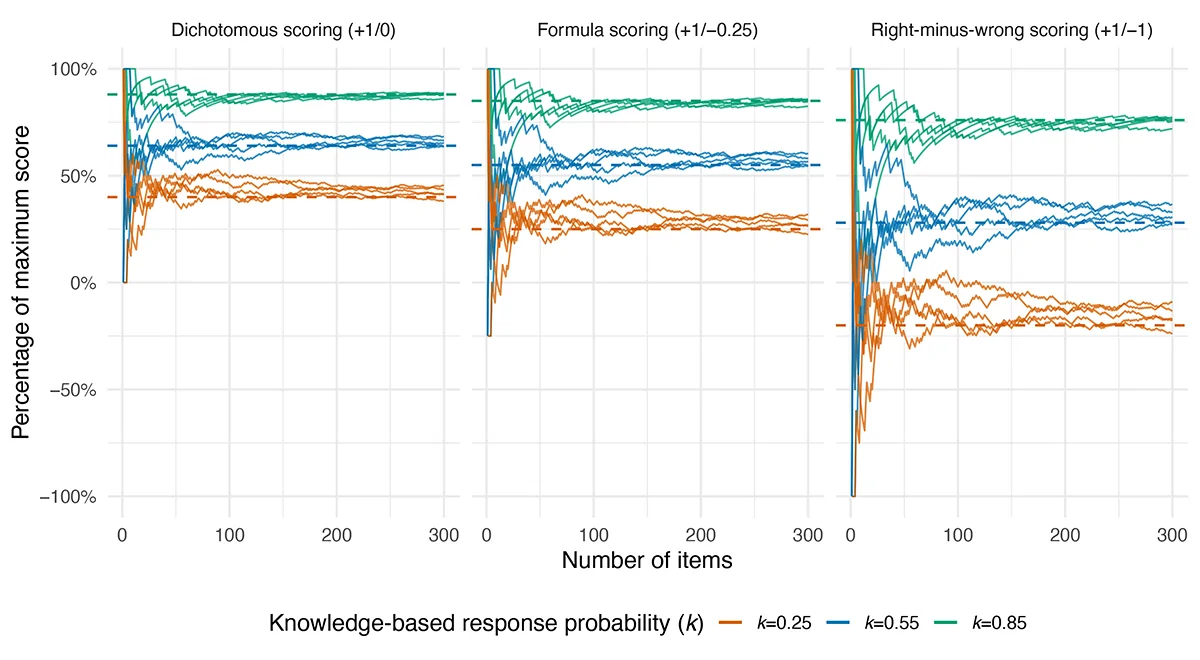
Convergence of examination result trajectories (percentage of the maximum possible score) with increasing item numbers. From a simulated cohort, 5 exemplary examinees were selected for different knowledge-based response probabilities (*k*=0.25, 0.55, and 0.85) and plotted for each scoring method. Dashed lines indicate the expected score for each *k*.

### Effects of Varying Scoring Methods

As shown in Figure 2, expressing results as a percentage of the maximum possible score yields a linear rescaling of the score scale across different scoring methods. Although the scale is transformed, the ordering of examinees and the position of the pass mark remain unchanged when thresholds are aligned. Accordingly, the magnitude of observed fluctuations is stretched or compressed, whereas the shape of score trajectories with increasing item numbers remains unchanged across scoring methods. If pass marks are defined consistently with the respective scoring scale (ie, using the score threshold corresponding to the same model-defined cutoff), the resulting pass or fail classifications are identical across all tested scoring methods. This finding also held across all modeled test lengths and distributions of *k*.

### Classification Accuracy and Knowledge Distribution

At the cohort level, overall classification accuracy (ie, correct pass or fail classification relative to the model-defined cutoff) increased with the number of items (Figure 3). Accuracy estimates became more stable with increasing cohort size: 95% simulation-based variability bands were wide for small cohorts (n=50), narrower for moderate cohorts (n=100), and narrow for large cohorts (n=1000), whereas the underlying classification accuracy itself remained unchanged. This pattern was consistent across both underlying distributions of *k* (uniform over [0,1] and truncated normal). The mean classification accuracy, however, differed by distribution. Under a uniform distribution, *k* is uniformly distributed, whereas the truncated normal distribution results in a higher concentration of examinees in the vicinity of the cutoff region, where discrimination between passing and failing is at its lowest and misclassification risk is at its highest. Consequently, cohort-level accuracy increased more slowly under the truncated normal distribution. For the uniform distribution, mean accuracy reached 0.90 after 24 items and 0.95 after 94 items (Figure 3A). In contrast, under a normal distribution truncated to [0,1] and centered at *k*=0.5 (SD 0.45/1.96; approximately 95% of *k* values fell between 0.05 and 0.95), mean accuracy reached 0.90 after 71 items and approached 0.95 only marginally at 300 items (Figure 3B). These item numbers are model- and distribution-dependent and should not be interpreted as recommended examination lengths. Similar plots for sensitivity and specificity, as well as the underlying data for accuracy, sensitivity, and specificity are shown in Multimedia Appendices 4–6.

**Figure 3.**
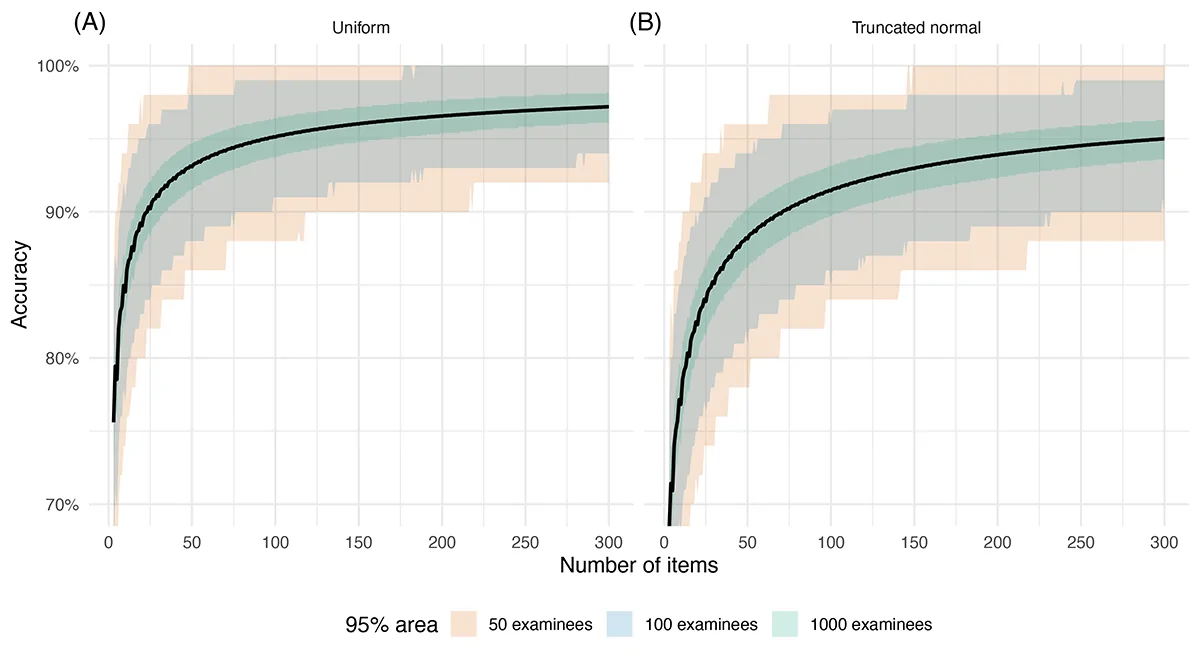
Cohort-level classification accuracy according to the number of items, with variability bands depending on cohort size. Classification accuracy and variability bands were calculated based on 10,000 repetitions of simulations of 50, 100, or 1000 examinees with knowledge-based response probabilities following (A) a uniform distribution or (B) a truncated normal distribution over the interval [0,1].

### Misclassification Rates by Knowledge-Based Response Probability Level

Misclassification rates were estimated using repeated simulations at fixed *k* levels (Table 2). For each *k* level, cohorts of 1000 examinees were simulated, with 10,000 repetitions per level. Misclassifications were strongly concentrated around the cutoff and declined rapidly as knowledge-based response probability moved away from the decision boundary. For very low *k* (eg, *k*≤0.2), incorrect classifications fell below 0.1% by 50 items and approached zero beyond 100 items. For high *k* (eg, *k*≥0.85), incorrect classifications were already rare at 20 items and approached zero thereafter. In contrast, misclassification risk remained substantially elevated near the cutoff even at large item counts. At *k*=0.45 (ie, fail), the mean misclassification rate was 9% at 300 items. At *k*=0.55 (ie, pass), it remained 6.7% at 300 items. At the exact cutoff (*k*=0.5), the expected observed score coincides with the classification boundary. Consequently, increasing test length does not lead to near-certain classification. At 300 items, approximately 47.5% of simulated examinees were classified as failing despite the model-defined reference classification of passing (Table 2).

**Table 2.** Incorrect classification results by knowledge-based response probability and number of items.

Knowledge-based response probability	True classification	Number of items (%^a^)							
		10	20	30	50	100	150	200	300
0.05	Fail	1.61	0.06	<0.01	<0.01	<0.01	<0.01	<0.01	<0.01
0.10	Fail	3.43	0.28	0.03	<0.01	<0.01	<0.01	<0.01	<0.01
0.15	Fail	6.38	0.92	0.15	<0.01	<0.01	<0.01	<0.01	<0.01
0.20	Fail	10.73	2.47	0.63	0.05	<0.01	<0.01	<0.01	<0.01
0.25	Fail	16.63	5.65	2.13	0.34	<0.01	<0.01	<0.01	<0.01
0.30	Fail	24.09	11.22	5.74	1.66	0.09	0.01	<0.01	<0.01
0.35	Fail	32.9	19.76	12.88	5.97	1.06	0.21	0.04	<0.01
0.40	Fail	42.71	31.28	24.45	16.08	6.62	2.97	1.39	0.32
0.45	Fail	53.01	45.02	40.14	33.65	24.08	18.31	14.27	9.02
0.50	Pass	36.69	40.44	42.14	43.9	45.68	46.46	46.92	47.49
0.55	Pass	27.08	26.81	25.61	22.89	17.41	13.48	10.59	6.72
0.60	Pass	18.66	15.66	12.92	8.82	3.61	1.55	0.69	0.14
0.65	Pass	11.81	7.83	5.17	2.33	0.34	0.06	0.01	<0.01
0.70	Pass	6.7	3.2	1.54	0.38	0.01	<0.01	<0.01	<0.01
0.75	Pass	3.27	1	0.32	0.03	<0.01	<0.01	<0.01	<0.01
0.80	Pass	1.3	0.21	0.04	<0.01	<0.01	<0.01	<0.01	<0.01
0.85	Pass	0.37	0.03	<0.01	<0.01	<0.01	<0.01	<0.01	<0.01
0.90	Pass	0.06	<0.01	<0.01	<0.01	<0.01	<0.01	<0.01	<0.01
0.95	Pass	<0.01	<0.01	<0.01	<0.01	<0.01	<0.01	<0.01	<0.01

a Values are mean percentages across 10,000 repeated simulations per *k* level, each simulation comprising a cohort of 1000 examinees. The model-defined cutoff was *k*=0.5.

If a fixed 60% pass mark is applied across all scoring methods (ie, without calibrating pass marks to the same underlying knowledge-based response probability threshold), misclassification rates follow a similar pattern. For formula scoring and right-minus-wrong scoring, examinees are required to show a higher level of knowledge-based response probability to achieve scores at or above the 60% pass mark (ie, 60% and 75%, respectively). Therefore, misclassifications cluster around these values of *k,* as shown in Multimedia Appendices 7 and 8.

### Items Required for High-Probability Correct Classification

For individual examinees, the number of items required to achieve a given probability of correct classification was derived for different levels of *k* (Figure 4; Table 3). The required number of items depended nonlinearly on *k* and increased sharply near the cutoff (*k*=0.5). In this region, classification was highly sensitive to random score variation. Substantially fewer items were needed further away from the cutoff. For example, at *k*=0.25, 17 items were required to reach *P* value (correct classification) ≥.95, whereas at *k*=0.85, 3 items were sufficient (Table 3). Near the cutoff, the required number of items increased markedly (eg, *k*=0.45: 402 items; *k*=0.55: 370 items for *P*≥.95; Table 3).

**Figure 4.**
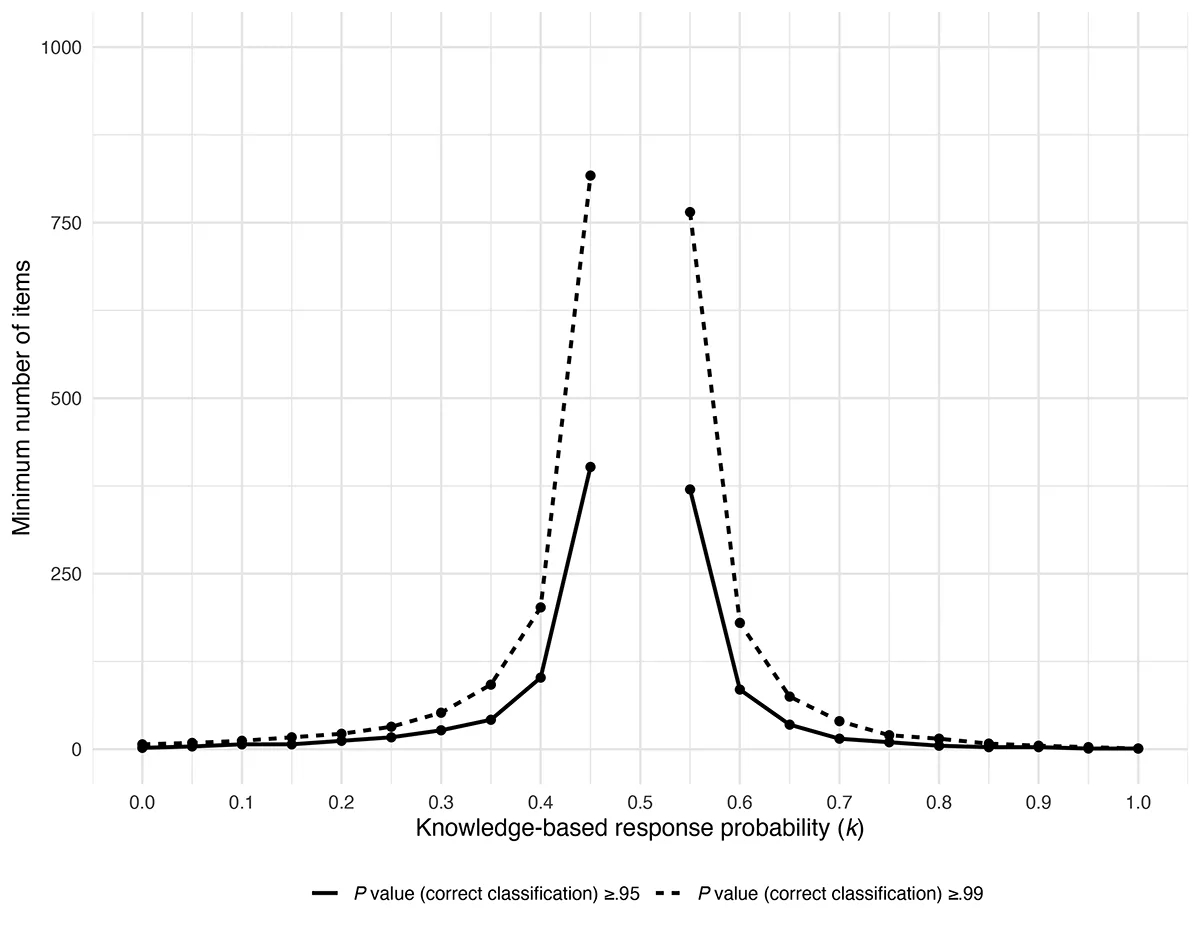
Model-based minimum test length for achieving a probability of correct pass or fail classification of ≥.95 or ≥.99 as a function of the knowledge-based response probability (*k*). The pass or fail cutoff was defined at *k*=0.5. Item numbers were limited to 1000 for graphical presentation.

**Table 3.** Minimum number of items required to achieve a high probability of correct classification for individual examinees.

Knowledge-based response probability	True classification	Items needed for *P* value (correct classification) ≥.95, n	Items needed for *P* value (correct classification) ≥.99, n
0.05	Fail	4	9
0.10	Fail	7	12
0.15	Fail	7	17
0.20	Fail	12	22
0.25	Fail	17	32
0.30	Fail	27	52
0.35	Fail	42	92
0.40	Fail	102	202
0.45	Fail	402	817
0.49	Fail	10,127	20,317
0.50	Pass	N/A^a^	N/A
0.51	Pass	9970	20,070
0.55	Pass	370	765
0.60	Pass	85	180
0.65	Pass	35	75
0.70	Pass	15	40
0.75	Pass	10	20
0.80	Pass	5	15
0.85	Pass	3	8
0.90	Pass	3	5
0.95	Pass	1	3

a N/A: not applicable (at *k*=0.5, the expected observed score coincides with the classification boundary; therefore, probabilities of correct classification of ≥.95 or ≥.99 cannot be attained by increasing test length).

## Discussion

### Principal Findings

This study demonstrates a fundamental invariance property of pass or fail classification decisions under linear score transformations, supported by analytical methods and simulations: under calibrated pass marks and assuming behavioral invariance, the investigated scoring methods for single-choice multiple-choice examinations yield identical classification outcomes. This equivalence follows mathematically from the linear relationship between the scoring rules; the simulations illustrate the effects of test length, distance from the cutoff, and the distribution of *k* on classification accuracy.

When decision thresholds are properly calibrated and examinees’ response behavior is not influenced by the scoring method, modifying scoring methods alone does not alter decision quality. These findings directly challenge the common assumption that penalty-based scoring methods improve the accuracy of pass or fail decisions.

### Structural vs Behavioral Effects of Scoring Methods

The present findings distinguish between two conceptually different mechanisms by which scoring methods may influence examination outcomes: (1) structural effects arising from mathematical score transformations and (2) behavioral effects arising from changes in examinees’ response behavior. By isolating the structural component, the present study shows that scoring methods alone do not change pass or fail classification outcomes when pass marks are aligned to the same model-defined cutoff. The absence of an effect under these conditions implies that scoring transformations do not improve classification accuracy independently of behavioral mechanisms. However, in real-world settings, scoring rules may influence examinees’ behavior, including omission strategies, guessing behavior, and risk-taking. Such behavioral responses may significantly affect observed scores independently of examinees’ knowledge-based response probability.

### Effects of Knowledge Distribution

Beyond scoring, classification accuracy increased with the number of items across all conditions. However, the rate of improvement depended strongly on the distribution of examinees’ knowledge-based response probability. Accuracy increased rapidly under a uniform distribution but substantially more slowly when examinees were concentrated around the cutoff. Misclassification risk was highest near the decision threshold and decreased as knowledge-based response probability moved away from this boundary. These patterns were identical across all scoring methods, indicating that classification accuracy is primarily determined by examinees’ *k* relative to the cutoff rather than by the scoring methods.

### Interpretation and Practical Implications

In practice, scoring rules may influence examinee behavior and thereby affect observed performance. Within the conditions simulated here, classification accuracy increased with test length. Penalty-based scoring methods merely rescale observed scores without changing the underlying classification when pass marks are calibrated, and response behavior remains unchanged. Additional concerns, such as construct-irrelevant variance related to risk-taking, increased test anxiety, fairness issues, and potential legal constraints [5-8], may further limit their practical justification in summative assessments where classification accuracy is the primary objective.

More broadly, these findings should be viewed in the context of longstanding efforts to correct multiple-choice scores for guessing [4,10,11]. From an educational perspective, the results indicate that classification accuracy in summative multiple-choice examinations is primarily determined by test length and the distribution of examinees’ knowledge-based response probability rather than by the choice of scoring method. Although scoring transformations modify the score scale, they do not alter the underlying decision problem when pass marks are aligned to the same model-defined cutoff. However, real-world assessment policy decisions may still need to consider behavioral responses of examinees and institutional or regulatory constraints.

Regarding pass or fail classifications in summative assessments, standard-setting procedures define minimum competence levels and are inherently affected by measurement error near the cutoff. Classification accuracy is lowest close to the decision threshold, where small variations in observed scores can lead to different classification outcomes [19-21]. In populations where many examinees are located near the pass or fail threshold, classification accuracy improves only slowly, even with large numbers of items. This pattern is consistent with established findings on decision accuracy and classification consistency in educational measurement [20,21] and emphasizes a fundamental limitation of dichotomous decision-making.

### Strengths and Limitations

A major strength of this study is the combination of analytical methods and large-scale Monte Carlo simulations. This allows for both exact and empirical evaluation of classification behavior under controlled conditions, and the calibrated pass marks ensured comparability across different scoring methods by focusing on the mathematical effects of score transformations. In addition, the systematic variation of item numbers, cohort sizes, and knowledge distributions provides a comprehensive characterization of classification accuracy across the prespecified range of testing scenarios.

The most important limitation of this study is the assumption of behavioral invariance. This assumption is intentionally restrictive and serves to isolate the purely mathematical contribution of scoring systems. In real-world examination settings, scoring rules can systematically influence examinees’ response behavior, including omission strategies, guessing behavior, and risk-taking [5-7]. Such behavioral adaptations may dominate observed differences between scoring methods and are not captured in the present model. The present findings should be interpreted as isolating the structural properties of scoring methods and not as a complete account of their impact in applied assessment contexts.

Beyond this central assumption, several additional modeling simplifications should be considered. First, item difficulty was not varied, implying a homogeneous item set and excluding potential effects of a realistic mix of easy and difficult questions or varying values of *k* across different topics on score convergence and classification performance. This simplification is intentional, as it isolates the effect of scoring transformations independently of item parameter heterogeneity, which would otherwise obscure the underlying invariance property. Second, informed guessing was not modeled, and responses were generated without incorporating partial knowledge or test-taking strategies such as systematic distractor elimination (ie, test-wiseness). Third, no item omissions were modeled, and all examinees were assumed to respond to all items. Fourth, the model assumes conditional independence between items, which may not hold in real examinations. Finally, although cohort size did not influence the classification mechanism itself, it affected the precision of estimated accuracy. In addition, the distribution of knowledge-based response probability in the target population is typically unknown but directly affects cohort-level classification accuracy.

Despite these simplifications, the model serves its intended purpose of isolating fundamental statistical relationships between scoring methods, test length, and classification accuracy.

### Future Directions

Future research should extend the present model to more realistic testing conditions. Incorporating heterogeneous item characteristics within an item response theory framework would allow the modeling of varying item difficulty and discrimination and provide a closer approximation to real examinations. In addition, future models should explicitly account for behavioral responses to different scoring methods, including omission strategies and individual differences in risk-taking, to assess the combined psychometric and behavioral effects of penalty-based scoring.

Further work also needs to model examinees’ partial knowledge and informed guessing, which may alter the relationship between *k* and observed response probabilities. Finally, empirical validation studies using real-world examination data are essential to determine the extent to which the theoretical equivalence of scoring methods observed under calibrated conditions holds in practice.

### Conclusions

When pass marks are calibrated to a common model-defined cutoff, the investigated scoring methods yield equivalent classification outcomes under complete responding and unchanged response behavior. Classification accuracy increased with test length and depended on the distribution of *k*, while uncertainty remained greatest near the cutoff. These findings describe the structural effects of the scoring transformations examined here and do not address behavioral effects or establish an optimal examination length.

## Supplementary material

10.2196/98358 Multimedia Appendix 1 Methods specification and reproducibility information describing the computational model, analytical derivations, simulation procedures, random-number settings, threshold and rounding conventions, and R code excerpts used for the reported analyses.

10.2196/98358 Multimedia Appendix 2 Machine-readable R data file containing the study-specific numerical outputs underlying the reported figures, tables, and supplementary analyses.

10.2196/98358 Multimedia Appendix 3 R session information documenting the software environment and package versions used for the final analyses.

10.2196/98358 Multimedia Appendix 4 Cohort-level sensitivity according to the number of items, with variability areas depending on cohort size. Sensitivity and variability areas were calculated based on 10,000 repetitions of simulations of 50, 100, or 1000 examinees with knowledge-based response probabilities following (A) a uniform distribution or (B) a truncated normal distribution over the interval [0,1].

10.2196/98358 Multimedia Appendix 5 Cohort-level specificity according to the number of items, with variability areas depending on cohort size. Specificity and variability areas were calculated based on 10,000 repetitions of simulations of 50, 100, or 1000 examinees with knowledge-based response probabilities following (A) a uniform distribution or (B) a truncated normal distribution (right) over the interval [0,1].

10.2196/98358 Multimedia Appendix 6 Overview of sensitivity, specificity, and accuracy according to the number of items and knowledge-based response probability following either a uniform distribution or a truncated normal distribution over the interval [0,1].

10.2196/98358 Multimedia Appendix 7 Visual representation of the misclassification rate for different scoring methods and varying levels of knowledge-based response probability and number of items if a fixed 60% pass mark is applied across all scoring methods (ie, pass marks are not calibrated to the same model-defined threshold).

10.2196/98358 Multimedia Appendix 8 Incorrect classification results by knowledge-based response probability and number of items if a fixed 60% pass mark is applied across all scoring methods (ie, pass marks are not calibrated to the same model-defined threshold).

## Abbreviations

FN: false negative
FP: false positive
TN: true negative
TP: true positive
