# Supplementary material for: Effects of Test Length and Scoring Methods on Pass or Fail Classification Accuracy in Single-Choice Examinations: Simulation Study
Source: JMIR Form Res. 2026 Oct 15;10:e98358. doi: 10.2196/98358 (PMC13671387; doi:10.2196/98358)
Supplement: Supplementary file 1 [file formative-v10-e98358-s004.pdf]

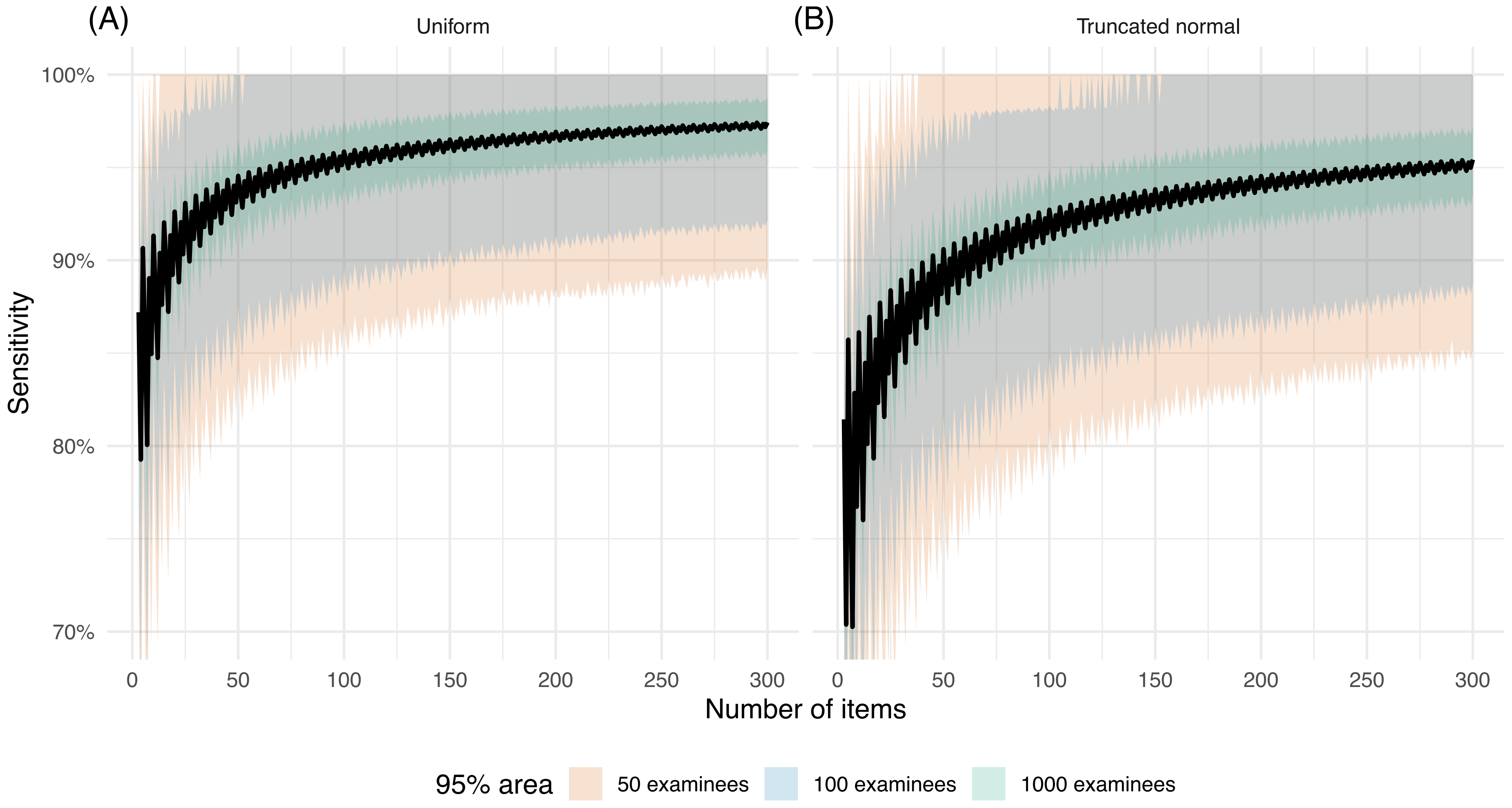
(A)
Uniform
(B)
Truncated normal
Sensitivity
100%
90%
80%
70%
0
50
100
150
200
250
300
Number of items
95% area
50 examinees
100 examinees
1000 examinees
