# Supplementary material for: Effects of Test Length and Scoring Methods on Pass or Fail Classification Accuracy in Single-Choice Examinations: Simulation Study
Source: JMIR Form Res. 2026 Oct 15;10:e98358. doi: 10.2196/98358 (PMC13671387; doi:10.2196/98358)
Supplement: Supplementary file 3 [file formative-v10-e98358-s007.pdf]

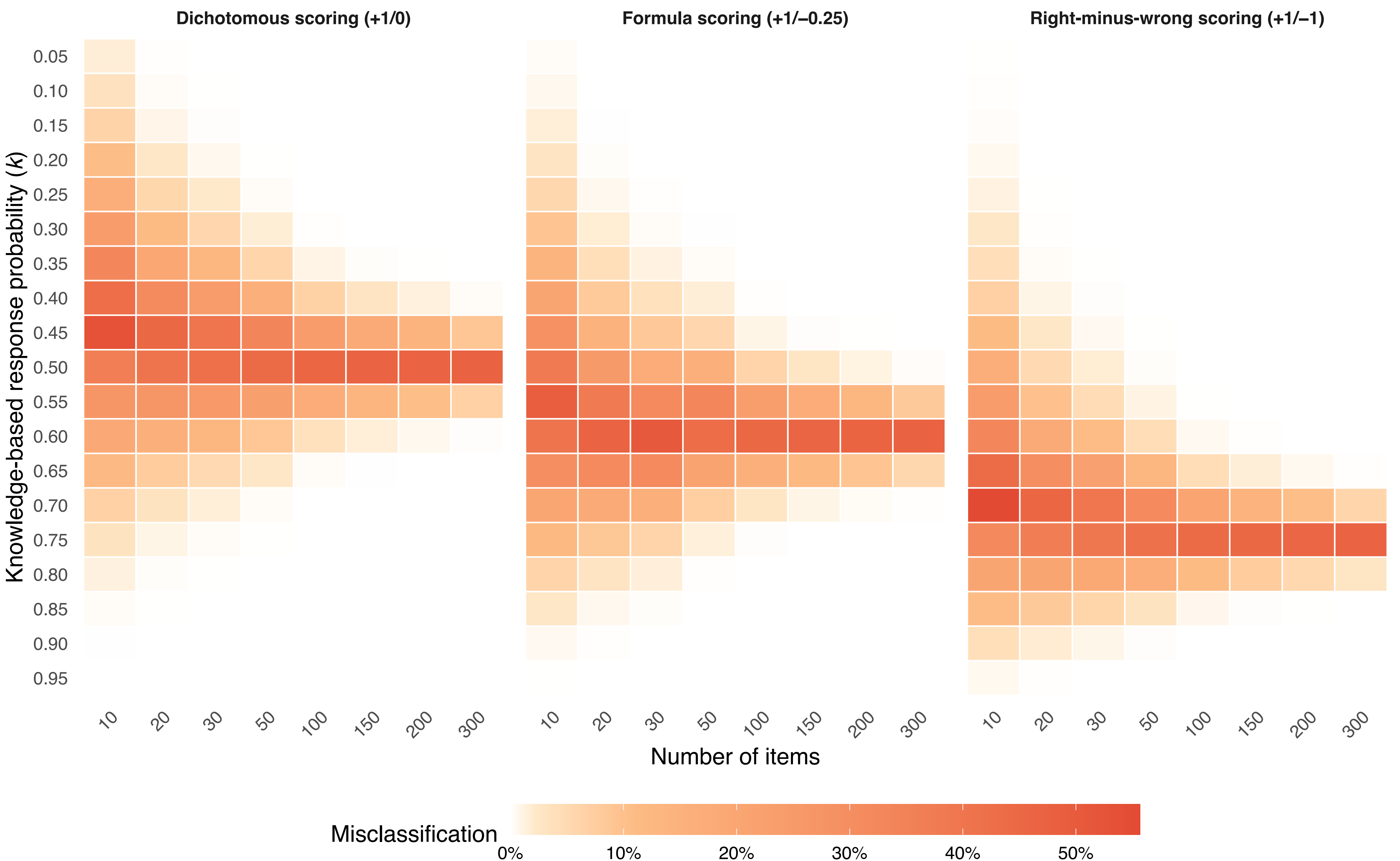

Dichotomous scoring (+1/0)
Formula scoring (+1/–0.25)
Right-minus-wrong scoring (+1/–1)
Knowledge-based response probability (k)
0.05
0.10
0.15
0.20
0.25
0.30
0.35
0.40
0.45
0.50
0.55
0.60
0.65
0.70
0.75
0.80
0.85
0.90
0.95
10
20
30
50
100
150
200
300
Number of items
Misclassification
0%
10%
20%
30%
40%
50%
