# Supplementary material for: Effects of Test Length and Scoring Methods on Pass or Fail Classification Accuracy in Single-Choice Examinations: Simulation Study
Source: JMIR Form Res. 2026 Oct 15;10:e98358. doi: 10.2196/98358 (PMC13671387; doi:10.2196/98358)
Supplement: Supplementary file 4 [file formative-v10-e98358-s001.docx]

Methods Specification and Reproducibility Information

**Effects of Test Length and Scoring Methods on Pass or Fail Classification Accuracy**

**in Single-Choice Examinations: Simulation Study**

**Purpose.** This document specifies the computational model, analytical derivations, Monte Carlo procedures, random-number settings, threshold and rounding conventions, and implementation details used for the study. Code excerpts are included to document the computational steps. Machine-readable study-specific simulation outputs underlying the reported figures and tables are supplied separately as an R data file (Multimedia Appendix 2), together with the R session information from the final analysis environment (Multimedia Appendix 3).

# 1. Software and computational environment

All analyses were conducted in R version 4.5.2. Data manipulation and graphics mainly used the packages dplyr, tidyr, purrr, ggplot2, and scales. Fixed random-number seeds were used for all stochastic components. The accompanying session-information file records the software environment and package versions used for the final analysis.

RStudio was used as the integrated development environment for code organization and execution.

For computationally intensive simulation steps, vectorized operations and direct sampling from the corresponding probability distributions were preferred over examinee- or item-wise loops whenever mathematically equivalent. This substantially reduces computation time and memory requirements in large Monte Carlo simulations. Iterative procedures were retained where sequential evaluation was inherent to the analysis, such as identifying the minimum test length required to reach a prespecified classification probability.

# 2. Response model

All modeled items were single-choice Type A items with one correct option and four distractors. Every item was answered. Each examinee was characterized by a knowledge-based response probability *k* ∈ [0,1]. The parameter *k* represents the assumed probability that a response is produced on the basis of knowledge rather than guessing (model parameter).

If an examinee responds on the basis of knowledge (probability *k*), the response is correct. Otherwise (probability 1 − *k*), the examinee guesses uniformly among the five answer options.

Therefore:

***p*(*k*) = *k* + (1 − *k*)/5 = 0.20 + 0.80*k***

```
n_options <- 5L
p_correct <- function(k) {
  k + (1 - k) / n_options
}
```

Conditional on *k*, responses to different items were assumed to be independent Bernoulli trials with success probability *p*(*k*). A response vector for one examinee can therefore be generated as follows:

k <- 0.55
m <- 300L
x <- rbinom(m, size = 1, prob = p_correct(k))

# 3. Scoring methods and calibrated pass marks

Three scoring rules were evaluated: dichotomous scoring, formula scoring, and right-minus-wrong scoring. Pass marks were calibrated to the expected score of an examinee with *k*=0.50, so that all three scoring scales represented the same model-defined classification boundary.

| **Scoring method** | **Correct response** | **Incorrect response** | **Pass mark at k=0.50** |
| --- | --- | --- | --- |
| Dichotomous scoring | +1 | 0 | 0.60 |
| Formula scoring | +1 | −0.25 | 0.50 |
| Right-minus-wrong scoring | +1 | −1 | 0.20 |

```
k_star <- 0.50
p_star <- p_correct(k_star)       # 0.60

PM_DS <- p_star                 # 0.60
PM_FS <- 1.25 * p_star - 0.25   # 0.50
PM_RW <- 2 * p_star - 1         # 0.20
```

Let *C* denote the number of correct responses in a test of *m* items. The three total scores are monotone affine transformations of *C*. Under complete responding and calibrated thresholds, they therefore yield identical (theoretical) pass or fail classifications:

score_DS <- C
score_FS <- 1.25 * C - 0.25 * m
score_RW <- 2 * C - m

pass_DS <- score_DS >= m * PM_DS
pass_FS <- score_FS >= m * PM_FS
pass_RW <- score_RW >= m * PM_RW

# 4. Pass or fail rule and attainable-score rounding

The model-defined reference classification was pass for *k*≥0.50 and fail for *k*<0.50. For a test containing *m* items, the calibrated dichotomous pass mark of 0.60 corresponds to the minimum attainable number of correct answers

***C*_min_(*m*) = ceiling(0.60 × *m*).**

Thus, when the nominal threshold is not an integer number of correct responses, it is rounded upward to the next attainable score. A small numerical tolerance may be applied solely to prevent floating-point artifacts when 0.60*m* is mathematically an integer.

```
required_correct <- function(m) {
  ceiling(0.60 * m - 1e-12)
}

# Example: 17 items
required_correct(17)   # 11 correct responses required
```

# 5. Exact analytical classification probabilities

For fixed *k* and *m*, the number of correct responses *C* follows a binomial distribution with parameters *m* and *p*(*k*). With *c*_min_=*C*_min_(*m*), the exact probability of passing is

***P*(pass | *k*,*m*) = *P*(*C* ≥ *c*_min_) = 1 − FBin(*c*_min_−1; *m*, *p*(*k*)).**

```
q_pass <- function(k, m) {
  cut <- required_correct(m)
  1 - pbinom(cut - 1, size = m, prob = p_correct(k))
}

p_correct_classification <- function(k, m) {
  qp <- q_pass(k, m)
  if (k >= 0.50) qp else 1 - qp
}
```

These exact binomial probabilities were used for the individual-level high-probability classification analysis (Table 3 and Figure 4) and as analytical checks for the simulation-based results. A normal approximation with continuity correction was used only as a secondary numerical check:

```
q_pass_normal_approx <- function(k, m) {
  p <- p_correct(k)
  cut <- required_correct(m)
  z <- (cut - 0.5 - m * p) / sqrt(m * p * (1 - p))
  1 - pnorm(z)
}
```

Table 3 was based exclusively on exact binomial probabilities; normal approximation was not used as a hybrid decision rule.

# 6. Random-number settings

Fixed seeds were set before independent stochastic analysis blocks to ensure reproducibility of the archived final outputs.

| **Analysis component** | **Seed** | **Role** |
| --- | --- | --- |
| Figures 1 and 2 | 1234 | Raw response simulation and selection of illustrative examinees |
| Table 2 | 123 | Repeated fixed-k cohort simulation |
| Figure 3 and Multimedia Appendices 4-6 | 123 | Repeated cohort simulations |
| Multimedia Appendices 7 and 8 | Deterministic | Exact binomial calculations; no random seed required |

```
set.seed(1234)  # illustrative trajectories
# ... simulation block ...

set.seed(123)   # principal repeated Monte Carlo analyses
# ... simulation block ...
```

# 7. Distribution of the knowledge-based response probability k

Two distributions of k were investigated: (1) a uniform distribution over [0,1], and (2) a normal distribution centered at 0.50 and truncated to [0,1]. The truncated-normal analyses used SD = 0.45/1.96 ≈ 0.22959, corresponding to an untruncated normal distribution with approximately 95% of values between 0.05 and 0.95.

```
sample_k_uniform <- function(n) runif(n, 0, 1)

sd_trunc <- 0.45 / 1.96

sample_k_truncnorm <- function(n, mean = 0.5, sd = sd_trunc,
                               low = 0, high = 1) {
  out <- numeric(0)
  while (length(out) < n) {
    need <- n - length(out)
    cand <- rnorm(max(2 * need, 200), mean = mean, sd = sd)
    cand <- cand[cand >= low & cand <= high]
    out <- c(out, cand)
  }
  out[seq_len(n)]
}
```

The truncated distribution was generated by rejection sampling: values outside [0,1] were discarded and resampled until the requested cohort size was reached.

# 8. Illustrative individual score trajectories (Figures 1 and 2)

Figures 1 and 2 are illustrative graphical representations of score variation across increasing test length. Five examinees were selected at each of *k*=0.25, 0.55, and 0.85 from a simulated cohort, and the same underlying correct/incorrect responses were transformed under all three scoring rules.

```
# Example for one examinee
k <- 0.55
m <- 300L
x <- rbinom(m, 1, p_correct(k))

score_DS <- x
score_FS <- ifelse(x == 1, 1, -0.25)
score_RW <- ifelse(x == 1, 1, -1)

cum_DS <- cumsum(score_DS)
cum_FS <- cumsum(score_FS)
cum_RW <- cumsum(score_RW)

prop_DS <- cum_DS / seq_len(m)
prop_FS <- cum_FS / seq_len(m)
prop_RW <- cum_RW / seq_len(m)
```

The machine-readable source data supplied with the study contain the selected trajectories used in the published figures, allowing the figures to be regenerated without repeating the random selection step.

# 9. Fixed-*k* misclassification analysis (Table 2)

Table 2 evaluates *k*=0.05, 0.10, …, 0.95 at test lengths *m*=10, 20, 30, 50, 100, 150, 200, and 300. For every (*k*,*m*) cell, 10,000 independent cohorts of 1,000 examinees were simulated. Because all examinees in a given cell share the same *k* and *m*, the number passing in a cohort can be generated directly from a binomial distribution with success probability *P*(pass|*k*,*m*). This is probabilistically equivalent to simulating 1,000 independent pass or fail outcomes after integrating over item responses for the same fixed *k*.

```
R_rep <- 10000L
n_per_cohort <- 1000L
k <- 0.45
m <- 100L

qp <- q_pass(k, m)
pass_n <- rbinom(R_rep, size = n_per_cohort, prob = qp)

misclassification <- if (k < 0.50) {
  pass_n / n_per_cohort
} else {
  1 - pass_n / n_per_cohort
}

mean(misclassification)
```

For validation, the simulation mean was compared with the corresponding exact misclassification probability, i.e., q_pass(*k*,*m*) for *k*<0.50 and 1−q_pass(*k*,*m*) for *k*≥0.50.

# 10. Cohort-level accuracy, sensitivity, and specificity (Figure 3 and Multimedia Appendices)

For each simulated cohort, model-defined status was based on *k*≥0.50 and observed status on the calibrated pass threshold. True positives (TP), false negatives (FN), true negatives (TN), and false positives (FP) were then used to calculate sensitivity, specificity, and accuracy. Figure 3 and the supplementary sensitivity/specificity analyses used 10,000 repeated cohorts. Simulation-based variability areas were defined by the empirical 2.5th and 97.5th percentiles across repeated cohorts of *n*=50, *n*=100, and *n*=1,000 examinees.

```
# One cohort at one test length; uniform distribution shown as an example
n <- 1000L
m <- 100L
k <- sample_k_uniform(n)
true_pass <- k >= 0.50

n_correct <- rbinom(n, size = m, prob = p_correct(k))
observed_pass <- n_correct >= required_correct(m)

TP <- sum(observed_pass &  true_pass)
FN <- sum(!observed_pass & true_pass)
TN <- sum(!observed_pass & !true_pass)
FP <- sum(observed_pass & !true_pass)

sensitivity <- TP / (TP + FN)
specificity <- TN / (TN + FP)
accuracy    <- (TP + TN) / n
```

A repeated-cohort analysis applies the same calculation independently across replications and test lengths. Mean curves are the arithmetic means of the cohort-level metrics at each test length; the variability limits are empirical quantiles across replications. The archived R data file contains the final study-specific mean curves and variability limits used in the published graphics.

```
# Generic summarization of repeated cohort-level values
mean_value <- mean(metric_values, na.rm = TRUE)
limits <- quantile(metric_values, probs = c(0.025, 0.975),
                   na.rm = TRUE, type = 8)
```

# 11. Minimum item numbers for high-probability correct classification (Table 3 and Figure 4)

For each prespecified *k* and target probability (0.95 or 0.99), the minimum test length was defined as the smallest integer *m* for which the exact binomial probability of correct classification reached the target. Table 3 required searches beyond 1,000 items for values close to the cutoff; Figure 4 was graphically limited to 1,000 items.

```
minimum_items <- function(k, target, m_max = 30000L) {
  if (abs(k - 0.50) < 1e-12) return(NA_integer_)

  for (m in seq_len(m_max)) {
    if (p_correct_classification(k, m) >= target) return(m)
  }
  NA_integer_
}

minimum_items(0.45, 0.95)  # 402
```

At *k*=0.50, the expected response rate equals the classification boundary. Consequently, probabilities of correct classification of 0.95 or 0.99 cannot be attained by increasing test length; Table 3 therefore reports *NA* for *k*=0.50.

# 12. Fixed 60% pass-mark supplementary analysis (Multimedia Appendices)

A supplementary analysis applied a fixed pass mark of 60% of the maximum possible score to all three scoring methods rather than calibrating each pass mark to the common *k*=0.50 boundary. Under this fixed score threshold, the implied *k* boundaries are 0.50 for dichotomous scoring, 0.60 for formula scoring, and 0.75 for right-minus-wrong scoring.

score_DS <- function(c_prop) c_prop
score_FS <- function(c_prop) 1.25 * c_prop - 0.25
score_RW <- function(c_prop) 2 * c_prop - 1

pass_DS <- score_DS(c_prop) >= 0.60
pass_FS <- score_FS(c_prop) >= 0.60
pass_RW <- score_RW(c_prop) >= 0.60

For a given scoring rule and test length, the fixed 60% score threshold can be translated into the corresponding minimum attainable number of correct responses. Exact binomial pass and misclassification probabilities were then evaluated across the reported *k* levels and test lengths.

```
cut_count <- function(m, scheme) {
  if (scheme == "DS") return(ceiling(0.60 * m - 1e-12))
  if (scheme == "FS") return(ceiling(0.68 * m - 1e-12))
  if (scheme == "RW") return(ceiling(0.80 * m - 1e-12))
  stop("Unknown scoring scheme")
}
```

# 13. Machine-readable study outputs

The accompanying R data file contains the study-specific numerical objects underlying the reported figures and tables. These archived outputs permit direct inspection of the numerical results and regeneration of the published displays without rerunning the Monte Carlo generation step.

The archived material comprises:

- source data for the individual trajectories shown in Figures 1 and 2
- cohort-level accuracy curves and simulation-based variability limits for Figure 3
- corresponding sensitivity and specificity results for Multimedia Appendices 4 and 5
- the numerical accuracy, sensitivity, and specificity table for Multimedia Appendix 6
- fixed-*k* misclassification results underlying Table 2
- minimum item numbers underlying Table 3 and Figure 4
- fixed-60% supplementary misclassification results underlying Multimedia Appendices 7 and 8
- the study parameters required to interpret these objects

The R data file can be loaded and inspected using standard R commands:

load("98358-Multimedia-Appendix-2.RData")
ls()
str(object_name)
head(object_name)

The archived values are study-specific outputs rather than raw item-response data. Figures and tables can be regenerated from these source objects using standard R data manipulation and plotting procedures.

# 14. Numerical validation

The final analyses were cross-checked against analytical results and the archived study outputs. Monte Carlo estimates were consistent with the corresponding exact probabilities within expected simulation error, and all reported numerical results and plotted series were verified against the machine-readable source data.

# 15. Model scope

Our model isolates structural effects of scoring transformations under complete responding and unchanged response behavior. It does not incorporate item-specific difficulty or discrimination, informed guessing, partial knowledge, omissions, distractor elimination, strategic responding, fatigue, content coverage, or behavioral changes induced by penalty scoring. Accordingly, the reported results quantify properties of the specified model and should not be interpreted as direct estimates of real-world examinee competence.

# 16. Reproducibility statement

Detailed model specifications, analytical equations, fixed random-number settings, threshold and rounding conventions, and selected implementation code are provided in this supplementary document. Machine-readable study-specific simulation outputs underlying the reported tables and figures are provided separately as an R data file, together with the software-session information from the final analysis environment. These materials permit independent verification of the reported calculations and regeneration of the published numerical and graphical results.
