# Supplementary material for: Effects of Test Length and Scoring Methods on Pass or Fail Classification Accuracy in Single-Choice Examinations: Simulation Study
Source: JMIR Form Res. 2026 Oct 15;10:e98358. doi: 10.2196/98358 (PMC13671387; doi:10.2196/98358)
Supplement: Supplementary file 5 [file formative-v10-e98358-s006.docx]

**Multimedia Appendix 6:** Overview of sensitivity, specificity, and accuracy according to the number of items and knowledge-based response probability following either a uniform distribution or a truncated normal distribution over the interval [0,1].

| **Number of items** | **Knowledge distribution** | **Sensitivity** | **Specificity** | **Accuracy** |
| --- | --- | --- | --- | --- |
| 10 | Uniform | 0.9131 | 0.7774 | 0.8452 |
| 10 | Truncated normal | 0.8613 | 0.6757 | 0.7685 |
| 20 | Uniform | 0.9262 | 0.8549 | 0.8905 |
| 20 | Truncated normal | 0.8770 | 0.7684 | 0.8227 |
| 30 | Uniform | 0.9348 | 0.8865 | 0.9106 |
| 30 | Truncated normal | 0.8893 | 0.8124 | 0.8508 |
| 50 | Uniform | 0.9456 | 0.9160 | 0.9308 |
| 50 | Truncated normal | 0.9058 | 0.8572 | 0.8815 |
| 100 | Uniform | 0.9585 | 0.9437 | 0.9511 |
| 100 | Truncated normal | 0.9273 | 0.9018 | 0.9145 |
| 150 | Uniform | 0.9651 | 0.9551 | 0.9601 |
| 150 | Truncated normal | 0.9383 | 0.9211 | 0.9297 |
| 200 | Uniform | 0.9691 | 0.9617 | 0.9654 |
| 200 | Truncated normal | 0.9454 | 0.9323 | 0.9389 |
| 300 | Uniform | 0.9742 | 0.9694 | 0.9718 |
| 300 | Truncated normal | 0.9542 | 0.9454 | 0.9498 |
