# Supplementary material for: Effects of Test Length and Scoring Methods on Pass or Fail Classification Accuracy in Single-Choice Examinations: Simulation Study
Source: JMIR Form Res. 2026 Oct 15;10:e98358. doi: 10.2196/98358 (PMC13671387; doi:10.2196/98358)
Supplement: Supplementary file 6 [file formative-v10-e98358-s008.docx]

**Multimedia Appendix 8:** Incorrect classification results by knowledge-based response probability and number of items if a fixed 60 pass mark is applied across all scoring methods (ie, pass marks are not calibrated to the same model-defined threshold).

| ***Knowledge-based response probability*** | **Scheme** | **Number of items (%^a^)** | | | | | | | |
| --- | --- | --- | --- | --- | --- | --- | --- | --- | --- |
|  |  | **10** | **20** | **30** | **50** | **100** | **150** | **200** | **300** |
| 0.05 | DS^b^ | 1.61 | 0.06 | <0.01 | <0.01 | <0.01 | <0.01 | <0.01 | <0.01 |
| 0.05 | FS^c^ | 0.27 | <0.01 | <0.01 | <0.01 | <0.01 | <0.01 | <0.01 | <0.01 |
| 0.05 | RW^d^ | 0.03 | <0.01 | <0.01 | <0.01 | <0.01 | <0.01 | <0.01 | <0.01 |
| 0.10 | DS | 3.42 | 0.27 | 0.02 | <0.01 | <0.01 | <0.01 | <0.01 | <0.01 |
| 0.10 | FS | 0.70 | 0.01 | <0.01 | <0.01 | <0.01 | <0.01 | <0.01 | <0.01 |
| 0.10 | RW | 0.10 | <0.01 | <0.01 | <0.01 | <0.01 | <0.01 | <0.01 | <0.01 |
| 0.15 | DS | 6.37 | 0.91 | 0.15 | <0.01 | <0.01 | <0.01 | <0.01 | <0.01 |
| 0.15 | FS | 1.55 | 0.06 | <0.01 | <0.01 | <0.01 | <0.01 | <0.01 | <0.01 |
| 0.15 | RW | 0.25 | <0.01 | <0.01 | <0.01 | <0.01 | <0.01 | <0.01 | <0.01 |
| 0.20 | DS | 10.72 | 2.47 | 0.63 | 0.05 | <0.01 | <0.01 | <0.01 | <0.01 |
| 0.20 | FS | 3.05 | 0.21 | 0.02 | <0.01 | <0.01 | <0.01 | <0.01 | <0.01 |
| 0.20 | RW | 0.59 | <0.01 | <0.01 | <0.01 | <0.01 | <0.01 | <0.01 | <0.01 |
| 0.25 | DS | 16.62 | 5.65 | 2.12 | 0.34 | <0.01 | <0.01 | <0.01 | <0.01 |
| 0.25 | FS | 5.48 | 0.65 | 0.09 | <0.01 | <0.01 | <0.01 | <0.01 | <0.01 |
| 0.25 | RW | 1.23 | 0.03 | <0.01 | <0.01 | <0.01 | <0.01 | <0.01 | <0.01 |
| 0.30 | DS | 24.07 | 11.23 | 5.74 | 1.66 | 0.09 | <0.01 | <0.01 | <0.01 |
| 0.30 | FS | 9.08 | 1.72 | 0.36 | 0.05 | <0.01 | <0.01 | <0.01 | <0.01 |
| 0.30 | RW | 2.36 | 0.11 | <0.01 | <0.01 | <0.01 | <0.01 | <0.01 | <0.01 |
| 0.35 | DS | 32.88 | 19.77 | 12.86 | 5.96 | 1.06 | 0.21 | 0.04 | <0.01 |
| 0.35 | FS | 14.10 | 3.97 | 1.24 | 0.34 | <0.01 | <0.01 | <0.01 | <0.01 |
| 0.35 | RW | 4.20 | 0.35 | 0.03 | <0.01 | <0.01 | <0.01 | <0.01 | <0.01 |
| 0.40 | DS | 42.70 | 31.27 | 24.45 | 16.09 | 6.62 | 2.97 | 1.39 | 0.32 |
| 0.40 | FS | 20.67 | 8.14 | 3.53 | 1.61 | 0.09 | <0.01 | <0.01 | <0.01 |
| 0.40 | RW | 7.02 | 0.96 | 0.15 | <0.01 | <0.01 | <0.01 | <0.01 | <0.01 |
| 0.45 | DS | 53.04 | 45.01 | 40.14 | 33.65 | 24.11 | 18.30 | 14.26 | 9.02 |
| 0.45 | FS | 28.77 | 14.99 | 8.52 | 5.71 | 0.95 | 0.18 | 0.03 | <0.01 |
| 0.45 | RW | 11.11 | 2.33 | 0.54 | 0.03 | <0.01 | <0.01 | <0.01 | <0.01 |
| 0.50 | DS | 36.69 | 40.44 | 42.15 | 43.90 | 45.67 | 46.46 | 46.93 | 47.50 |
| 0.50 | FS | 38.23 | 25.00 | 17.63 | 15.61 | 6.15 | 2.65 | 1.19 | 0.25 |
| 0.50 | RW | 16.73 | 5.10 | 1.72 | 0.22 | <0.01 | <0.01 | <0.01 | <0.01 |
| 0.55 | DS | 27.08 | 26.83 | 25.59 | 22.89 | 17.39 | 13.48 | 10.59 | 6.72 |
| 0.55 | FS | 48.68 | 38.03 | 31.58 | 33.36 | 23.44 | 17.50 | 13.42 | 8.24 |
| 0.55 | RW | 24.05 | 10.11 | 4.67 | 1.11 | 0.04 | <0.01 | <0.01 | <0.01 |
| 0.60 | DS | 18.67 | 15.68 | 12.92 | 8.82 | 3.61 | 1.56 | 0.69 | 0.14 |
| 0.60 | FS | 40.44 | 46.93 | 50.60 | 43.28 | 45.23 | 46.10 | 46.62 | 47.24 |
| 0.60 | RW | 33.13 | 18.27 | 10.97 | 4.37 | 0.54 | 0.07 | 0.01 | <0.01 |
| 0.65 | DS | 11.81 | 7.84 | 5.18 | 2.32 | 0.35 | 0.06 | <0.01 | <0.01 |
| 0.65 | FS | 29.79 | 31.69 | 31.90 | 21.30 | 15.80 | 11.95 | 9.17 | 5.55 |
| 0.65 | RW | 43.78 | 30.19 | 22.38 | 13.37 | 4.40 | 1.60 | 0.60 | 0.09 |
| 0.70 | DS | 6.70 | 3.20 | 1.55 | 0.38 | 0.01 | <0.01 | <0.01 | <0.01 |
| 0.70 | FS | 20.12 | 18.38 | 16.20 | 7.18 | 2.62 | 1.01 | 0.40 | 0.07 |
| 0.70 | RW | 55.58 | 45.61 | 39.61 | 31.78 | 20.82 | 14.60 | 10.55 | 5.79 |
| 0.75 | DS | 3.28 | 1.00 | 0.31 | 0.03 | <0.01 | <0.01 | <0.01 | <0.01 |
| 0.75 | FS | 12.09 | 8.67 | 6.11 | 1.44 | 0.16 | 0.02 | <0.01 | <0.01 |
| 0.75 | RW | 32.22 | 37.04 | 39.30 | 41.64 | 44.05 | 45.13 | 45.78 | 46.55 |
| 0.80 | DS | 1.30 | 0.21 | 0.04 | <0.01 | <0.01 | <0.01 | <0.01 | <0.01 |
| 0.80 | FS | 6.14 | 3.04 | 1.50 | 0.14 | <0.01 | <0.01 | <0.01 | <0.01 |
| 0.80 | RW | 20.64 | 20.59 | 19.36 | 16.61 | 11.21 | 7.72 | 5.40 | 2.72 |
| 0.85 | DS | 0.37 | 0.02 | <0.01 | <0.01 | <0.01 | <0.01 | <0.01 | <0.01 |
| 0.85 | FS | 2.39 | 0.67 | 0.19 | <0.01 | <0.01 | <0.01 | <0.01 | <0.01 |
| 0.85 | RW | 10.87 | 8.27 | 6.06 | 3.25 | 0.73 | 0.17 | 0.04 | <0.01 |
| 0.90 | DS | 0.06 | <0.01 | <0.01 | <0.01 | <0.01 | <0.01 | <0.01 | <0.01 |
| 0.90 | FS | 0.58 | 0.06 | <0.01 | <0.01 | <0.01 | <0.01 | <0.01 | <0.01 |
| 0.90 | RW | 4.01 | 1.83 | 0.82 | 0.17 | <0.01 | <0.01 | <0.01 | <0.01 |
| 0.95 | DS | <0.01 | <0.01 | <0.01 | <0.01 | <0.01 | <0.01 | <0.01 | <0.01 |
| 0.95 | FS | 0.04 | <0.01 | <0.01 | <0.01 | <0.01 | <0.01 | <0.01 | <0.01 |
| 0.95 | RW | 0.62 | 0.10 | 0.01 | <0.01 | <0.01 | <0.01 | <0.01 | <0.01 |

^a^Values are mean percentages across 10,000 repeated simulations per *k* level, each simulation comprising a cohort of 1000 examinees. The model-defined cutoff was *k*=0.5.

^b^Dichotomous scoring.

^c^Formula scoring.

^d^Right-minus-wrong scoring.
